# Intestinal transcriptomes in Kazakh sheep with different haplotypes after experimental *Echinococcus granulosus* infection

**DOI:** 10.1051/parasite/2021011

**Published:** 2021-03-05

**Authors:** Xin Li, Song Jiang, Xuhai Wang, Bin Jia

**Affiliations:** 1 College of Life Sciences, Shihezi University Road Beisi Shihezi 832003 Xinjiang PR China; 2 College of Animal Science and Technology, Shihezi University Road Beisi Shihezi 832003 Xinjiang PR China

**Keywords:** Differentially expressed genes, Disease resistance, *Echinococcus granulosus*, Intestinal tissue, Transcriptome analysis

## Abstract

Cystic echinococcosis (CE) is a chronic zoonosis caused by infection with the larval stage of the cestode *Echinococcus granulosus*. As the intermediate host, sheep are highly susceptible to this disease. Our previous studies have shown that sheep with haplotype *MHC Mva* Ibc-*Sac* IIab-*Hin*1I ab were resistant to CE infection, while their counterparts without this haplotype were not. In order to reveal the molecular mechanism of resistance in Kazakh sheep, after selecting the differential miRNA in our previous study, herein, transcriptome analyses were conducted to detect the differential expression genes in the intestinal tissue of Kazakh sheep with resistant and non-resistant MHC haplotypes, after peroral infection with *E. granulosus* eggs. A total of 3835 differentially expressed genes were identified between the two groups, with 2229 upregulated and 1606 downregulated. Further function analysis showed that the most significant genes were related to both innate immune response and adaptive response participating in the defense against *E*. *granulosus* infection and the metabolic changes associated with it. The results suggest that genes related to lectin receptors, NK cells activation, chemokines, and tumor necrosis factor, may play important roles in the response of intestinal tissue to *E. granulosus.*

## Abstract (fr)

L’échinococcose kystique (EK) est une zoonose chronique qui est causée par une infection au stade larvaire du cestode *Echinococcus granulosus*. En tant qu’hôte intermédiaire, les moutons sont très sensibles à cette maladie. Nos études précédentes ont montré que les moutons avec l’haplotype *MHC Mva Ibc-Sac IIab-Hin1I ab* étaient résistants à l’EK, alors que leurs homologues sans cet haplotype ne l’étaient pas. Afin de révéler le mécanisme moléculaire de la résistance chez le mouton kazakh, après avoir sélectionné le miARN différentiel dans notre étude précédente, des analyses de transcriptome ont été menées dans ce travail pour détecter les gènes d’expression différentielle dans le tissu intestinal de mouton kazakh avec des haplotypes MHC résistants et non résistants, après une infection pérorale par des œufs d’*E. granulosus*. Un total de 3835 gènes différentiellement exprimés ont été identifiés entre les deux groupes, avec 2229 régulés à la hausse et 1606 à la baisse. Une analyse fonctionnelle plus poussée a montré que les gènes les plus significatifs étaient liés à la fois à la réponse immunitaire innée et à la réponse adaptative participant à la défense contre l’infection à *E. granulosus* et aux changements métaboliques qui y étaient associés. Les résultats suggèrent que les gènes liés aux récepteurs de la lectine, à l’activation des cellules NK, aux chimiokines et au facteur de nécrose tumorale peuvent jouer un rôle important dans la réponse du tissu intestinal à *E. granulosus*.

## Introduction

Cystic echinococcosis (CE), a zoonotic disease caused by *Echinococcus granulosus*, poses a great threat to the health of humans and domestic animals [51]. The life cycle of *E. granulosus* is complex and involves two hosts: definitive and intermediate. Infection with *E. granulosus* occurs after oral ingestion of infective eggs (an oncosphere containing the invasive hexacanth [9]). The hexacanth then hatches in the small intestine of the intermediate host, penetrates the mucosal tissue, enters the blood circulation system, and migrates to various organs (e.g. the liver and lungs), where it develop cysts filled with fluid and protoscoleces [6]. However, in definitive hosts like dogs, adult parasites develop from the protoscolex in the intestine. In domestic animals, especially in sheep, which appear to be highly susceptible to infection [38], CE causes considerable health problems and thereby significantly affects the income of herders [32].

*Echinococcus granulosus* infection in the host is a complex dynamic process, which involves the recognition and interaction of a variety of biological molecules, including genes [33], proteins [1], and miRNAs [42]. Upregulation or downregulation of molecular expression likely causes different individuals of the same host to have different resistance, similarly to reports about *Plasmodium homocircumflexum* [17]. It is very important to understand the interaction mechanisms between the host and the parasite. Research and discussions about these mechanisms will help to determine the key factors of immune regulation in the host from the perspective of molecular biology, and further explore the mechanism of disease resistance. This research will also provide new ideas for mining molecular markers of disease resistance and directional genetic breeding [19].

Previously, we carried out a series of studies to research the relationship between MHC polymorphisms and resistance or susceptibility to CE in native Chinese sheep, including Chinese Merino [21], Duolang [28], and Kazakh sheep [29]. Our previous studies indicated that in Kazakh sheep, when exposed to the same number of parasites, the rate of *E. granulosus* infection in the internal organs of sheep with haplotype *MHC Mva*I bc-*Sac*II ab-*Hin*1I ab haplotype was significantly lower than in sheep without this haplotype. Statistical results and analysis showed that sheep with this haplotype have an increased genetic capability to respond to and subsequently reject *E. granulosus* when infected. The haplotype *MHC Mva*I bc*-Sac*II ab*-Hin*1I ab was therefore identified as a genetic marker of CE resistance in Kazakh sheep [29].

Afterwards, studies were conducted to reveal the mechanism of CE resistance in Kazakh sheep, and most of our research was focused on intestinal tissue. It has been found that when sheep were infected with oncospheres (larvae of *E. granulosus*), the small intestine tissue was the first mucosal immune barrier that must be passed through, where various antibodies, cytokines, and other immune molecules are expressed in response to exogenous stimuli [12]. These molecules could be involved in stopping larval growth of *E. granulosus* at the very first stages [11, 21, 28, 29]. Hence, we were interested to see the molecular mechanisms of CE resistance in Kazakh sheep with the resistant haplotype, at the very first stage of infection. Against this background, we previously performed microRNA sequencing, and found several microRNAs in the intestinal tissue, which were related to the inflammatory pathway and played important roles in CE resistance in sheep [22]. Since microRNAs exert their functions by regulating the expression of target genes, we were curious to see whether genes related to inflammation and immune response were varied in these sheep, including innate immune cells activation, chemokines, and tumor necrosis factor. It is, therefore, necessary to conduct genome-wide characterization of transcriptome profiles involved in resistance to CE infection in sheep, especially in sheep with different CE resistance.

Transcriptome sequencing aims at the set of all transcripts, including mRNAs, rRNA, tRNA, and other noncoding RNAs, produced in one or a population of cells in a specific developmental stage or physiological condition [13]. In the present study, using high-throughput transcriptome sequencing, we screened a number of differential genes in the small intestinal tissue of Kazakh sheep after infection with *E. granulosus*. In terms of their functions, we concluded that innate immune response plays the first defense role in CE resistance in sheep. In addition, adaptive immune response played an important role in defense against *E. granulosus* infection. Moreover, our study revealed that metabolic changes contribute to defense against *E. granulosus* infection. These findings laid the foundation for a discussion of the molecular mechanism of Kazakh sheep resistance to *E. granulosus*.

## Materials and methods

### Ethics statement

This study was performed in strict compliance with the recommendations of the National Institutes of Health guideline for the care and use of laboratory animals (NIH Publication No. 8023, revised 1978), and the Animal Care and Use Committee of Shihezi University approved all procedures and experiments (Approval No. A2018-138-01). Local strain dogs and Kazakh sheep were purchased from farmers around Shihezi city. The farmers gave oral consent for the use of their animals in this research after they were provided with explanations about the purpose of the research and on efforts to ensure the welfare of animals.

### Animals and experimental design

The preparation method of the experimental materials was similar to previous research reports [22]. Since no studies have shown that the CE resistance of sheep is related to sex, 2-year-old Kazakh ewes with CE-resistant [*Mva*I bc-*Sac*II ab-*Hin*1I ab(R)] and CE-non-resistant [*Mva*Ibc-*Sac*IIab-*Hin*1Iab (NR)] haplotypes were selected, as described previously, and raised on the farm of Shihezi University. All animals were raised under the same conditions of free access to water and food, in natural lighting. They were divided into two groups according to their MHC haplotype: three sheep with the CE-resistant haplotype were referred to as the CE-R group, three sheep with the CE-non-resistant haplotype constituted the CE-NR group, and a single sheep with the CE-non-resistant haplotype formed the CK (Control Check) group. All the sheep were screened for the absence of hydatid cysts and were healthy prior to the experiment. In line with the previous approach, all the sheep were orally infected with *E. granulosus* eggs. Since our previous research showed that there were already significant differences in miRNAs in the small intestine at 8 h after infection, we speculated that differential gene expression may occur at the same time point. As a result, all animals were sacrificed 8 h after infection. Immediately afterward, the intestinal tissue of every sheep was removed. Approximately 100-mm^3^-sized intestinal tissue blocks were collected and frozen immediately in liquid nitrogen prior to long-term storage at −80 °C until RNA extraction.

### Experimental pipeline of RNA-seq and quality control

Total RNA was isolated from every sample using TRIZOL reagent, and divided into two parts, one for RNA-seq, and the other for real-time PCR to verify the expression of genes detected by RNA-seq.

During the RNA-seq process, after DNase I treatment, magnetic beads with Oligo (dT) were used to isolate mRNA. Mixed with the fragmentation buffer, the mRNA was fragmented into short fragments. Then, cDNA was synthesized using mRNA fragments as templates. cDNA fragments were purified and resolved with EB buffer for end reparation and adenine addition. After that, the fragments were connected to adapters. After agarose gel electrophoresis, the suitable fragments were selected for the polymerase chain reaction (PCR) amplification as templates. During the quality control (QC) steps, an Agilent 2100 Bioanalyzer and ABI StepOnePlus Real-Time PCR System were used for the quantification and qualification of the sample library. In this process, the intestinal tissue of every sheep in the CE-R group was prepared to an individual paired-end library for RNA-seq, like in the CE-NR group. Each library was sequenced using Illumina HiSeq 2000 (BGI, Huada Genomics Institute, Shenzhen, China).

Primary sequencing data produced using Illumina HiSeq 2000, called raw reads, were filtered with the *SOAPnuke* program (version 1.5.0) [27] by (1) removing reads containing sequencing adapters; (2) removing reads whose low quality base ratio (base quality less than or equal to 5) was more than 20%; and (3) removing reads whose unknown base (“N” base) ratio was more than 5%, after clean reads were obtained and stored in FASTQ format. After the above quality control (QC) step, raw reads were filtered into clean reads. Because the CE-R group contained the clean data from three individual libraries (for three samples), these data were combined to form the clean data of the CE-R group, and were aligned to the reference genome with *SOAPaligner/SOAP2* [26], like in the CE-NR group. In this search, the sheep oar3.1 database of livestock genomics was selected as the reference genome (http://www.livestockgenomics.csiro.au/sheep/oar3.1.php/).

The clean data were used to calculate the distribution of reads on reference genes and perform coverage analysis. Gene coverage was calculated as the percentage of a gene covered by reads. This value is equal to the ratio of the base number in a gene covered by unique mapping reads to the total base number of that gene by the SAMtools program.

### Normalization of expressed genes

There are many different patterns to normalize the gene expression level. In this search, we used the *RPKM* method [36] (Reads per kilobase transcriptome per million mapped reads), because it is able to eliminate the influence of different gene lengths and sequencing discrepancy on the calculation of gene expression. Therefore, the calculated results can be directly used to compare the differences in gene expression between samples.

### Screening and verification of differentially expressed genes (DEGs)

After the alignment result passed QC, according to the significance of digital gene expression profiles which were reported on genome research by Audio [2], only genes with a value of FDR ≤ 0.001 and |log2 ratio| ≥ 1 were selected as DEGs. Since DEG analysis generated a large number of problems in which thousands of hypotheses (is gene *x* differentially expressed between the two groups) were tested simultaneously, correction for false-positive (type I errors) and false-negative (type II) errors was performed using the False Discovery Rate (FDR) method [4]. We assumed that we picked out *R* differentially expressed genes in which S genes actually showed differential expression and the other *V* genes were false positives. As we decided that the error ratio “*Q* = *V*/*R*” must stay below a cutoff, we preset the FDR to a number no larger than 0.001.

In addition, before transcriptome functional annotation, nine differentially expressed genes were randomly selected to conduct real-time PCR analysis to verify the expression of genes detected by RNA sequence. The *GAPDH* gene was used as an internal control (for PCR verifications only). Primer sequences are listed in Table 1. The mRNA expression levels for all samples were normalized to the level for *GAPDH* housekeeping genes. The expression level in every sample group was expressed by the mean ± standard deviation (*x* ± s). The data were analyzed by *SPSS*17.0 software.

**Table 1:** Primer sequences for qRT-PCR analysis of gene transcripts.

Gene	Primer sequences (5′–3′)	Expected size (bp)	Annealing temperature (°C)
*NTNG1*	F: CAAAGACAGGTTCGCATT	214	59.5
	R: CCTTCCGTGCACTTTTAT		
*EphA3*	F: CCAGCGATGTATGGAGTTA	180	58.5
	R: CTTTCTGCCAGCAGTCTAG		
*NCOA1*	F: GATCTAACCTCCCTTCTGCTTTT	228	61
	R: GGAACAGAGAGGACAACGCA		
*SCD*	F: CGCTGGCACATCAACTTTACC	124	56.6
	R: TTTCCTCTCCAGTTCTTTTCATCC		
*GHR*	F: AGGTTGCTCAGCCACAAA	281	58.9
	R: TGGGGAAAGGACCACATT		
*SMAD3*	F: CGACTACAGCCATTCCATCC	120	60.5
	R: TGGTCACTGGTCTCTCCATCT		
*TGFBR1*	F: ATTCTGTGGCTTTGCCTGAA	175	60
	R: GCAGTTTCCTGGGTCTGAAG		
*IGF1*	F: GACAGGAATCGTGGATGAGTG	270	57
	R: AACAGGTAACTCGTGCAGAGC		
*LPL*	F: GTCATCGTGGTGGACTGG	398	59.3
	R: TGGAAAGTGCCTCCGTTA		
*GAPDH*	F: CTGACCTGCCGCCTGGAGAAA	149	59.0
	R: GTAGAAGAGTGAGTGTCGCTGTT		

### Functional annotation

Gene Ontology (GO) enrichment analysis provides all GO terms that significantly enriched in a list of DEGs, compared to a genome background, and filter the DEGs that correspond to specific biological functions. All DEGs between two groups were mapped to GO terms in the database (http://www.geneontology.org/), calculating gene numbers for every term. We then used hypergeometric tests to find significantly enriched GO terms in the input list of DEGs, based on the GO:TermFinder program (http://smd.stanford.edu/help/GO-TermFinder/GO_TermFinder_help.shtml/). GO terms fulfilling a threshold of *p*-value ≤ 0.05 were defined as significantly enriched GO terms in DEGs. This analysis was able to recognize the main biological functions that DEGs exercise.

Functional annotations of the DEGs were conducted using the Blast2GO program against the public the NCBI nr database. The Kyoto Encyclopedia of Genes and Genomes (KEGG, http://www.genome.jp/kegg/) library was used to classify and group these identified DEGs.

## Results

In the present study, firstly, RNA-sequence analysis showed that 46,934,632 reads were obtained in the CE-R group, 76.60% of which matched the reference genome. The number of unique match reads was 30,693,890, accounting for 65.40% of the total reads. Similarly, 47,203,918 reads were obtained in the CE-NR group, with 81.71% matching to the reference genome. The number of unique match reads was 27,695,773, accounting for 58.67% of the total. Detailed information is listed in Table 2.

**Table 2:** Alignment statistics of reads and reference genome.

Alignment types	CE-R	CE-NR	CK
Total reads	46934632	47203918	47241022
Total base pairs	4224116880	4248352620	4251691980
Total mapped reads	35950168(76.60%)	38570247 (81.71%)	35662569 (75.49%)
Perfect matched reads	24082846 (51.31%)	24897763 (52.75%)	21930554 (46.42%)
≤5 bp Mismatched reads	11867322 (25.28%)	13672484 (28.96%)	13732015 (29.07%)
Unique match	30693890 (65.40%)	27695773 (58.67%)	28046058 (59.37%)
Multi-position match	5256278 (11.20%)	10874474 (23.04%)	7616511 (16.12%)
Total unmapped reads	10984464 (23.40%)	8633671 (18.29%)	11578453 (24.51%)

**Figure 1:**
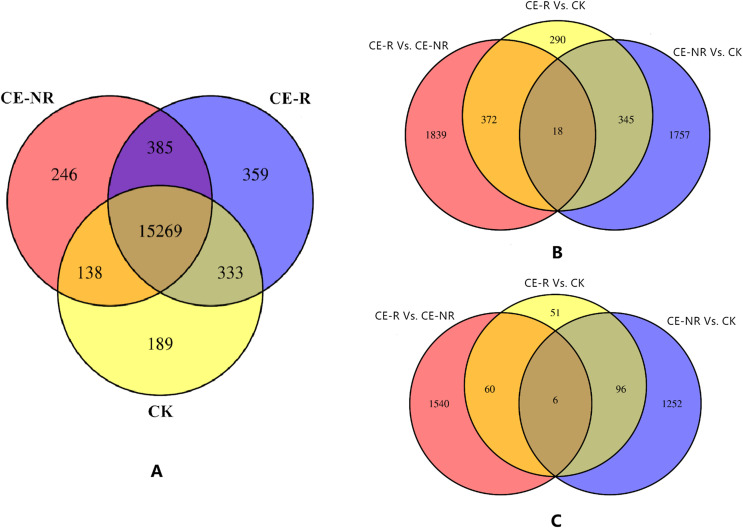
Venn diagram of all genes and differential expression genes (DEGs) between groups. Figure 1A shows all identified genes in each group. Figure 1B shows the upregulated DEGs between groups, while Figure 1C shows the downregulated DEGs between groups.

**Figure 2:**
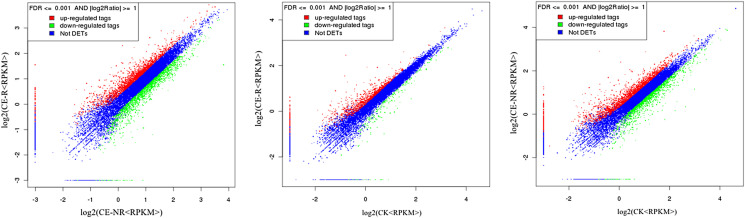
Scatter chart of differential expression genes between groups. Figure 2A shows differential expression genes between the CE-R and CE-NR groups. Figure 2B shows differential expression genes between the CE-R and CK groups. Figure 2C shows differential expression genes between the CE-NR and CK groups. *X*-axis and *Y*-axis presents two samples log2 value of gene expression, and the expression was calculated using the *RPKM* method (reads per kilobase transcriptome per million mapped reads) as shown in section “Materials and methods”, red (up) and green (down) dots mean the gene has significant differences (FDR ≤ 0.001, two fold difference), and the blue dot means no significant differences.

**Figure 3:**
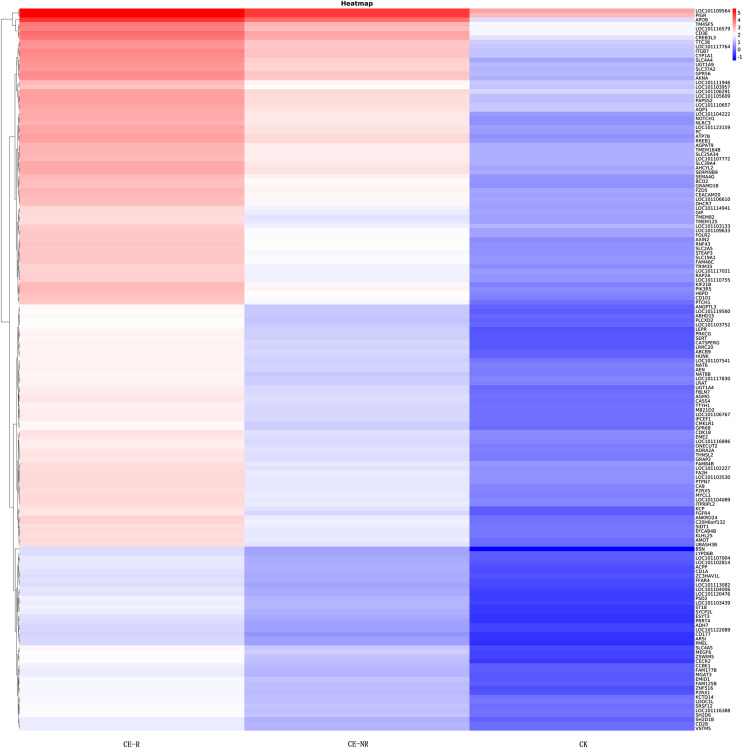
Heatmap of partial DEGs between sample groups.

Results showed that 16,346 genes were detected in CE-R group samples, while 16,038 were detected in the CE-NR group (Fig. 1A). Furthermore, the analysis of DEGs showed 3835 genes which were different in the CE-R group compared with CE-NR group, of which 2229 were upregulated and 1606 were downregulated. The Venn diagrams of DEGs between groups are shown in Figures 1B and 1C. Scatter charts of detected genes are shown in Figure 2, where the *X*-axis and *Y*-axis present two samples log2 value of expression, red (up) and green (down) dots mean the gene has significant differences (FDR ≤ 0.001, two fold difference), and the blue dot means no significant difference. A heatmap highlights the patterns of expression for many DEGs between two sample groups (Fig. 3).

In summary, GO functional clustering and KEGG pathway analysis showed that various DEGs related to immune response were significantly more highly expressed in the CE-R group than in the CE-NR group. Firstly, we found that some genes related to lectin receptors, including *CLEC9A*, *CLEC10A*, and *CLEC4G* were significantly more highly expressed in the CE-R group than in the CE-NR group. Secondly, we found that a large number of DEGs related to NK cell activation (*KLRC2*, *IRAK1*, *FCRL6*, and *NKG7*), mast cell activation (*MCP1* and *IRCP1*), and T cells were significantly more highly expressed in the CE-R group than in the CE-NR group. Additionally, genes related to chemokines (*CXCL12*, *CXCL14*, *CCL4*, *CCL5*, *CCL11*, *CCL25*, and *CCL28*), compensatory inflammation repair mechanisms (*CYP17A1*, *CYP1A1*, *CYP2C19*, and *CYP3A24*), and tumor necrosis factor (*TNFRSF13B*, *TNFRSF17*, *TNFRSF1A*, *TNFRSF4*, *TNFSF10*, *TNFSF13*, *TNFSF15*, *TNFSF18*) were more highly expressed in the CE-resistant group than in the CE-non-resistant sheep (Table 3). In addition, some genes related to MHC were also significantly more highly expressed in the CE-R group than in the CE-NR group, including *LOC101103957*, *LOC101108696*, *LOC101109747*, *LOC101120148*, *LOC101109080*, *LOC101105609* and *LOC101107908.*

**Table 3:** Partial differential expression genes in the intestine, organized according to their function group.

Gene	Gene ID	Description	Fold change between CE-R and CE-NR
Epithelial barrier-lectin secretion			
CLEC9A	101119033	C-type lectin domain containing 9A	11.73
CLEC10A	101117600	C-type lectin domain containing 10A	3.1
CLEC4G	101123627	C-type lectin domain containing 4G	8.13
NK cell activation			
IRAK1	101121405	Interleukin-1 receptor-associated kinase 1	3.24
NKG7	101115817	Natural killer cell granule protein 7	7.65
Mast cell activation and immune killing			
MCP1	443546	Monocyte chemoattractant protein-1	4.59
IRAK1	101121405	Interleukin-1 receptor-associated kinase 1	3.24
T lymphocyte activation			
NFATC2	101119239	Recombinant nuclear factor of activated T cells, cytoplasmic 2	5.38
NFAT5	101113952	Nuclear factor 5 of activated T cells	2.93
Chemokine expression			
CXCL12	101121145	Chemokine (C-X-C motif) ligand 12	3.42
CXCL14	101105243	Chemokine (C-X-C motif) ligand 14	4.61
CCL4	101114285	Chemokine (C-C motif) ligand 4	2.88
CCL5	101115553	Chemokine (C-C motif) ligand 5	10.24
CCL11	101119832	Chemokine (C-C motif) ligand 11	5.41
CCL25	678679	Chemokine (C-C motif) ligand 25	4.2
CCL28	780500	Chemokine (C-C motif) ligand 28	4.48
Promote white blood cell migration			
TICAM1	101111016	Toll like receptor adaptor molecule 1	3.82
Compensatory inflammation repair mechanism			
CYP17A1	493968	Cytochrome P450, family 17, subfamily A, polypeptide 1	15
CYP1A1	100170113	Cytochrome P450, family 1, subfamily A, polypeptide 1	5.9
CYP2C19	100534653	Cytochrome P450, family 2, subfamily C, polypeptide 19	11
CYP3A24	100170111	Cytochrome P450, family 3, subfamily A, polypeptide 24	7.91
TNF expression related			
TNFRSF13B	101122200	Tumor necrosis factor receptor superfamily member 13B	4.18
TNFRSF17	101111149	Tumor necrosis factor receptor superfamily member 17	14.75
TNFRSF1A	100135698	Tumor necrosis factor receptor superfamily member 1A	3.03
TNFRSF4	101108407	Tumor necrosis factor receptor superfamily member 4	2.88
TNFSF10	101114153	Tumor necrosis factor receptor superfamily member 10	3.36
TNFSF13	101123054	Tumor necrosis factor receptor superfamily member 13	3.13
TNFSF15	101102957	Tumor necrosis factor receptor superfamily member 15	2.97
TNFSF18	101108765	Tumor necrosis factor receptor superfamily member 18	5.15
MHC related			
LOC101103957	101103957	/	6.11
LOC101108696	101108696	/	4.45
LOC101109747	101109747	/	3.38
LOC101120148	101120148	/	3
LOC101109080	101109080	/	12.71
LOC101105609	101105609	/	6.35
LOC101107908	101107908	/	4.92

**Figure 4:**
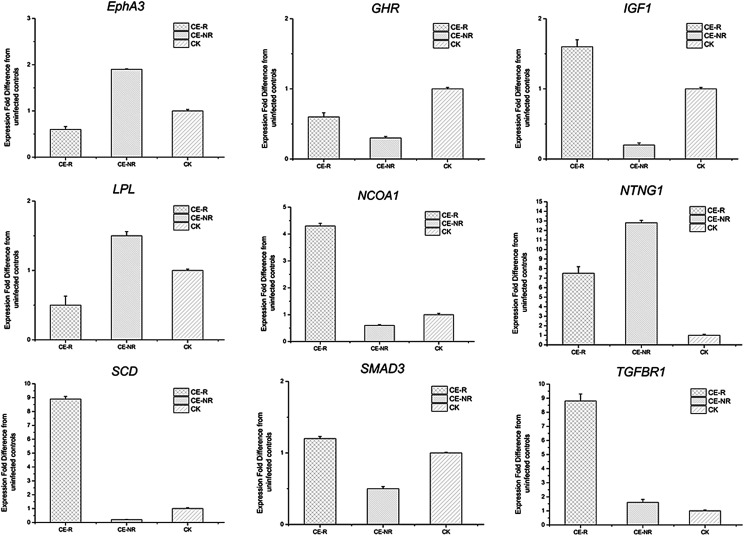
Validation of transcriptome data by qRT-PCR on nine randomly selected genes. Real-time PCR was employed to verify the regulation of genes detected by transcriptome. Nine differential expression genes were randomly selected. GAPDH was used as an internal control.

Furthermore, nine genes with a differential expression level were randomly selected to conduct real-time PCR validation of the transcriptome analysis. The results from real-time PCR are shown in Figure 4, and strongly correlated with those generated from transcriptome analysis.

## Discussion

Based on previous studies, we speculated that the small intestine of Kazakh sheep with different MHC genotypes was the first line of defense against *E. granulosus* infection, and the immune response mechanism in the intestine might be related to disease resistance. The experimental results confirmed our hypothesis that DEGs related to innate immune response and adaptive immune response were found in different groups of sheep, some of which are worthy of attention.

### Innate immune response plays the first defense role in CE resistance in sheep

The innate immune system is the first line of defense for the host to protect itself against invasion and infection by various parasites. During the infection process, as important recognition receptors, lectins can bind with carbohydrate molecules on the surface of parasites, initiate related immune response, kill and eliminate invasive parasites, and play an important role in innate immunity against parasite infection.

In this study, we found that when Kazakh sheep were infected with oncospheres, the expression of *CLEC9A*, *CLEC10A*, and *CLEC4G* genes was significantly higher in the CE-R group than in the CE-NR group. All these genes are C-type lectin receptor family members, which has been demonstrated for immunomodulatory effects against parasitic infections in the epithelial layer [45]. Reports have shown that upregulation of C-type lectin genes is beneficial to recognition of parasite antigens, thus activating antigen presentation more effectively [31], which has a positive effect on disease resistance [18, 49]. Based on these reports and our findings, we speculated that the high regulation of lectin receptor genes in the intestinal tissue may enhance the recognition of parasite antigens and present them to the internal immune cells, which can induce the immune system to kill the parasite. This effect was more significant in the CE-R sheep than in the CE-NR sheep, and it may lead to different *E. granulosus* resistance of Kazakh sheep with different MHC genotypes.

Furthermore, MHC class I chain-related protein A (MICA) is a natural ligand of C-type lectin like activated receptor, which is mainly expressed in the human intestinal epithelium. Cellular stress such as parasitic infections and inflammation can upregulate the expression of MICA [3]. However, although we found that *CLEC9A* and other genes were upregulated in CE-R group, no evidence showed corresponding MHC natural ligand. This should be studied further.

In addition to the antigen recognition system, innate immune cells, including natural killer (NK) cells [43], mast cells [5], and macrophages [8] can also be activated and produce immune response when host tissues are infected by parasites. Reports have shown that if the expression levels of genes related to the activation and receptor recognition of these cells are upregulated, this may promote phagocytosis [37], killing of parasites [43] and inflammatory response [20] of hosts, and result in stronger resistance to parasites.

Natural killer cells are responsible for activating the cytotoxic killing effect in infections, mainly to defend against early infections by viruses [34], bacteria [15], and parasites [41]. The killing effect of NK cells on target cells depends on intracellular interaction of activated and inhibitory receptors on the cell surface [10]. NKG7 (Natural killer cell granule protein 7) serves as an activating receptor for NK cells, which is associated with inflammatory diseases, since it could augment the production of inflammatory factors, including IFN-γ, responsible for type I immune responses [44]. In this study, the expression of *NKG7* was significantly higher in the CE-R group than in the CE-NR group, with a fold change of 7.65 (*p*-value: 3.38E-211, FDR: 1.04E-209). A current study by Ng (personal communication) showed that *NKG7* was one of the most upregulated genes from the livers of mice infected with *Leishmania donovani*. Results indicated that NKG7 regulated the proinflammatory responses effectively, and the survival of the parasite was obviously affected [39]. Given these reports, and the results of our study, we inferred that the high expression of *NKG7* in CE-R sheep is beneficial for the host to activate NK cells and kill parasites, which is beneficial for CE resistance.

Mast cells are widely distributed around the capillaries under the skin and visceral mucosa, secreting a variety of cytokines, which participate in immune regulation (TB cell and APC cell activation), and express MHC and B7 molecules [16]. In our study, we found that mast cell-related genes, including *FCRL6* and *IRAK-1*, were highly expressed in the CE-R group. *IRAK-1* is related to Toll-like receptors (TLRs). When oncospheres of the parasite infect the small intestinal tissue, various antigens secreted by the parasite can be recognized by mast cells through TLRs, which activate effective immune response and produce immediate inflammation. This can be evidenced by the upregulation of genes related to inflammation and chemokines.

### Adaptive immune response played an important role in defense against *E. granulosus* infection

It has been demonstrated that the mechanisms of host immune response to CE infection are complex. Response involves not only the innate immune system but also the adaptive immune system. In addition, we found that adaptive immune response played an important role in defense against *E. granulosus* infection. Nuclear factor of activated T cells (NFAT) could activate T cell response, thus the transcription of cytokines and other genes in immune response [24]. Besides T cells, NFAT is also expressed on many immune cells, such as B lymphocytes [23]. In this study, we found that the expression of *NFATC2* was higher in the CE-R group than in the CE-NR group, with a 5.38 fold change (*p*-value: 4.04E-51, FDR: 3.17E-50). Furthermore, the *NFAT5* was upregulated in the CE-R group, with a 2.93 fold change (*p*-value: 1.84E-27, FDR: 8.91E-27). Therefore, it was speculated that the high expression of *NFAT-*activated T cells, or expressed on B cells, induces adaptive immune response, and produces antibodies. As expected, in our study, we found that IgM and IgE were significantly more highly expressed in CE-resistant sheep than in non-resistant sheep.

### Metabolic changes during defense responses against *E. granulosus* infection in Kazakh sheep

Finally, our study revealed that cytochrome metabolism may contribute to defense against *E. granulosus* infection. KEGG pathway enrichment analysis showed that many DEGs associated with Cytochrome 450 (CYP450), including *CYP1A*, *CYP3A* and *CYP26A* in CE-resistant sheep were clearly higher than in CE-non-resistant sheep. These DEGs were mainly enriched in the “metabolism of xenobiotics by cytochrome P450” pathway (46 genes, *p-value*: 2.357955e-08; Pathway ID: ko00980) and “drug metabolism – cytochrome P450” pathway (39 genes, *p*-value: 5.695932e-08, Pathway ID: ko00982). The CYP450 enzymes family includes membrane-bound hemoproteins that play a pivotal role in the detoxification of xenobiotics, cellular metabolism, and homeostasis [14]. It has been reported that genes of the CYP450 family varied similarly in the liver of experimental mice infected with *E. multilocularis* [30]. Moreover, a recent study demonstrated that members of the CYP family CYP1A could enhance inflammatory responses [48]. This aligns with the results of our previous study showing several upregulated miRNAs, which are related to NF-KB inflammation pathways, and play important roles in the response of intestinal tissue to *E. granulosus* infection in sheep with resistant haplotypes [22]. We can infer that the upregulation of the CYP family in the present study might enhance inflammatory responses, thus contributing more strongly to defense responses to CE infection in CE-R sheep than in CE-NR sheep.

In addition, we found that retinol metabolism is associated with CE infection in sheep. After CE infection, 38 DEGs associated with retinol metabolism, including *ADH* [7], *RDH* [50], *UGT* [25], and *LRAT* [35], were significantly higher in CE-resistant sheep than in non-resistant sheep. These genes are already known to be associated with the binding, metabolic process, and transport of retinol. Reports have shown that retinol plays an important role in maintaining host immune function. Cotton rats lacking retinol had a significantly higher infection rate of filarial worms compared with normal cotton rats [46], and a similar finding was reported in research of human infection with filarial worms [40]. Parasites may take retinol preferentially from the host at the infected site for their own development, differentiation, growth, and reproduction. High concentrations of retinol in worms have a significant effect on their growth rate. Other studies have suggested that retinol is critical to defense against parasite infections, and this is due to the fact that a lack of retinol damages the epithelial tissue barrier and helps filariae to penetrate and migrate in the host, and local immune response of the mucosal surface thereby decreases [47]. Retinol is, therefore, vital in host resistance to parasitic infections. Based on these findings, we speculated that the high expression of these genes in the CE-R group might play an important role in intestinal defense against *E. granulosus* infection, by hindering parasite damage to the epithelial tissue barrier, as well as penetration and migration to the host organ. These types of inhibitory effects might be related to the artificial infection resistance in the small intestinal tissue of Kazakh sheep reported by us previously [4, 5]. This is the first finding of a relationship between retinol metabolism and CE resistance in the host.

In conclusion, we found a number of genes related to the resistance of Kazakh sheep to echinococcosis in the present study, mainly related to lectin secretion, immune cell activation, antigen binding, chemokine expression, and metabolic pathways like retinol. Further studies, like using RNAi or Crispr/cas9 to verify the function of these genes are still needed in the future. In addition to transcriptomics analysis, Multi-omics research including proteomics, metabolomics, and lipidomics is still needed to reveal the molecular mechanism of CE resistance in Kazakh sheep with the resistant haplotype. We are currently carrying out a proteomics study to select different proteins in the intestinal tissue of these Kazakh sheep. Combined with the results of this study, we hope future results will enable us to provide a comprehensive explanation of resistance to CE in Kazakh sheep.

## Conflict of interest

The authors declare that they have no conflicts of interest in this work: we declare that we do not have any commercial or associative interest that represents a conflict of interest in connection with the work submitted.
